# Cryo-Electron Microscopy Reveals That Sperm Modification Coincides with Female Fertility in the Mosquito *Aedes aegypti*

**DOI:** 10.1038/s41598-019-54920-6

**Published:** 2019-12-06

**Authors:** Jade M. Noble, Ethan C. Degner, Laura C. Harrington, Lena F. Kourkoutis

**Affiliations:** 1 Robert Frederick Smith School of Chemical and Biomolecular Engineering, Cornell University, Ithaca, New York USA; 2 Department of Entomology, Cornell University, Ithaca, New York USA; 3 Department of Entomology, Cornell University, Ithaca, New York USA; 4 School of Applied and Engineering Physics, Cornell University, Ithaca, New York USA; 5 Kavli Institute at Cornell for Nanoscale Science, Cornell University, Ithaca, New York USA

**Keywords:** Microscopy, Cryoelectron microscopy, Entomology, Structural biology, Cryoelectron microscopy, Animal physiology, Animal behaviour, Reproductive biology

## Abstract

Manipulating mosquito reproduction is a promising approach to reducing mosquito populations and the burden of diseases they carry. A thorough understanding of reproductive processes is necessary to develop such strategies, but little is known about how sperm are processed and prepared for fertilization within female mosquitoes. By employing cryo-electron microscopy for the first time to study sperm of the mosquito *Aedes aegypti*, we reveal that sperm shed their entire outer coat, the glycocalyx, within 24 hours of being stored in the female. Motility assays demonstrate that as their glycocalyx is shed in the female’s sperm storage organs, sperm transition from a period of dormancy to rapid motility—a critical prerequisite for sperm to reach the egg. We also show that females gradually become fertile as sperm become motile, and that oviposition behavior increases sharply after females reach peak fertility. Together, these experiments demonstrate a striking coincidence of the timelines of several reproductive events in *Ae. aegypti*, suggesting a direct relationship between sperm modification and female reproductive capacity.

## Introduction

Mosquito reproduction is a promising target for controlling populations of mosquitoes that transmit human disease. While traditional mosquito control often aims to limit breeding sites^1^ or kill larvae and adult mosquitoes with insecticides^2^, interfering with reproduction after insemination could be a more efficient strategy for population reduction. Several such strategies are being developed or implemented. For example, in the sterile insect technique, irradiated males are released to mate with wild females (which are normally monogamous^3–6^), but males transfer sperm incapable of fertilization (reviewed in^7^). A similar technique (being tested in the mosquitoes *Aedes aegypti* and *Ae. albopictus*) deploys males with *Wolbachia* bacterial endosymbionts, which, when mated with uninfected, wild females, prevent reproduction by inducing cytoplasmic incompatibility between sperm and egg (reviewed in^8,9^). Genetically modified males that produce inviable offspring have been deployed to successfully reduce local mosquito populations^10,11^. Finally, gene drive technologies are being developed to genetically sterilize females of the African malaria mosquito *Anopheles gambiae*^12^. Despite the promise of these vector control methods that manipulate post-copulatory reproductive processes, little is known about how sperm function in females after insemination.

A growing body of literature suggests that post-insemination modifications of sperm structure and motility are common phenomena across Animalia^13^. These modifications may serve to initiate or modify sperm motility, help sperm adapt to diverse environments encountered in the female reproductive tract, or prepare them for fertilization (reviewed in^13^). Sperm modifications are best described in mammals. For example, intracellular pH and calcium concentrations increase and ultimately lead to hyperactivated motility (reviewed in^14^), and in humans, certain components of the plasma membrane are rearranged to facilitate exocytosis during fertilization^15^. In the ostracod crustacean *Mytilocypris mytiloides*, sperm are initially equipped with two protective layers, but shed their outer coat once stored in the female^16^. The removal of this layer coincides with the onset of sperm motility and oviposition, suggesting that losing this coat may be required for female fertility. Similarly, eupyrene sperm (nucleated sperm that partake in fertilization) in the silkworm moth *Bombyx mori* also shed an outer sheath while in the female reproductive tract, whereas infertile apyrene sperm (without nuclei) do not. Because both sperm types are motile but sheath loss is specific to eupyrene sperm, it is inferred that loss of their outer sheath facilitates successful storage or fertilization^17^. Finally, micrographs of sperm of the mosquitoes *Culex quinquefasciatus*^18^ and *Toxorhynchites brevipalpis*^19^ suggest that removal of a glycocalyx, a cell coat containing a dense network of carbohydrate-rich molecules, occurs in these taxa as well, although it is unclear when such a removal occurs, what its purpose may be, or whether it is typical of all mosquito sperm.

Understanding when and where mosquito sperm are modified in the female reproductive tract may provide insight into how and why they are altered. Here, we describe the timing of sperm modification and processing in the mosquito *Ae. aegypti*—the primary vector of the viruses that cause dengue, yellow fever, and Zika. We used cryo-electron microscopy to investigate sperm ultrastructure before and at different times after insemination. This imaging technique allowed us to view mosquito sperm frozen in a near native state—without fixatives and stains that could alter their ultrastructure. We find that sperm are initially covered in a thick, highly organized glycocalyx, but within 12 h of storage within the female, most sperm lack this coat. Using motility and fertility experiments, we attempt to correlate changes in sperm ultrastructure to function. Between periods of rapid motility in the bursa (where sperm are initially deposited in the female) and later in the spermathecae (where sperm are held for long term storage), we describe a period of inactivity shortly after insemination. To understand whether female reproductive capacity changes as sperm are modified, we also investigated how soon after mating females are able to fertilize eggs and lay them. We find that females both gain fertility and are stimulated to lay eggs while sperm are shedding their glycocalyx and after sperm have regained rapid motility. Understanding the timing of physiological events is a crucial first step to identifying their underlying cellular and molecular mechanisms. Our results provide the foundation for future investigations of critical reproductive processes that could be manipulated for the purpose of vector control.

## Results

### Cryo-electron microscopy of mature *Ae. aegypti* sperm reveals known and novel ultrastructural detail

**Figure 1:**
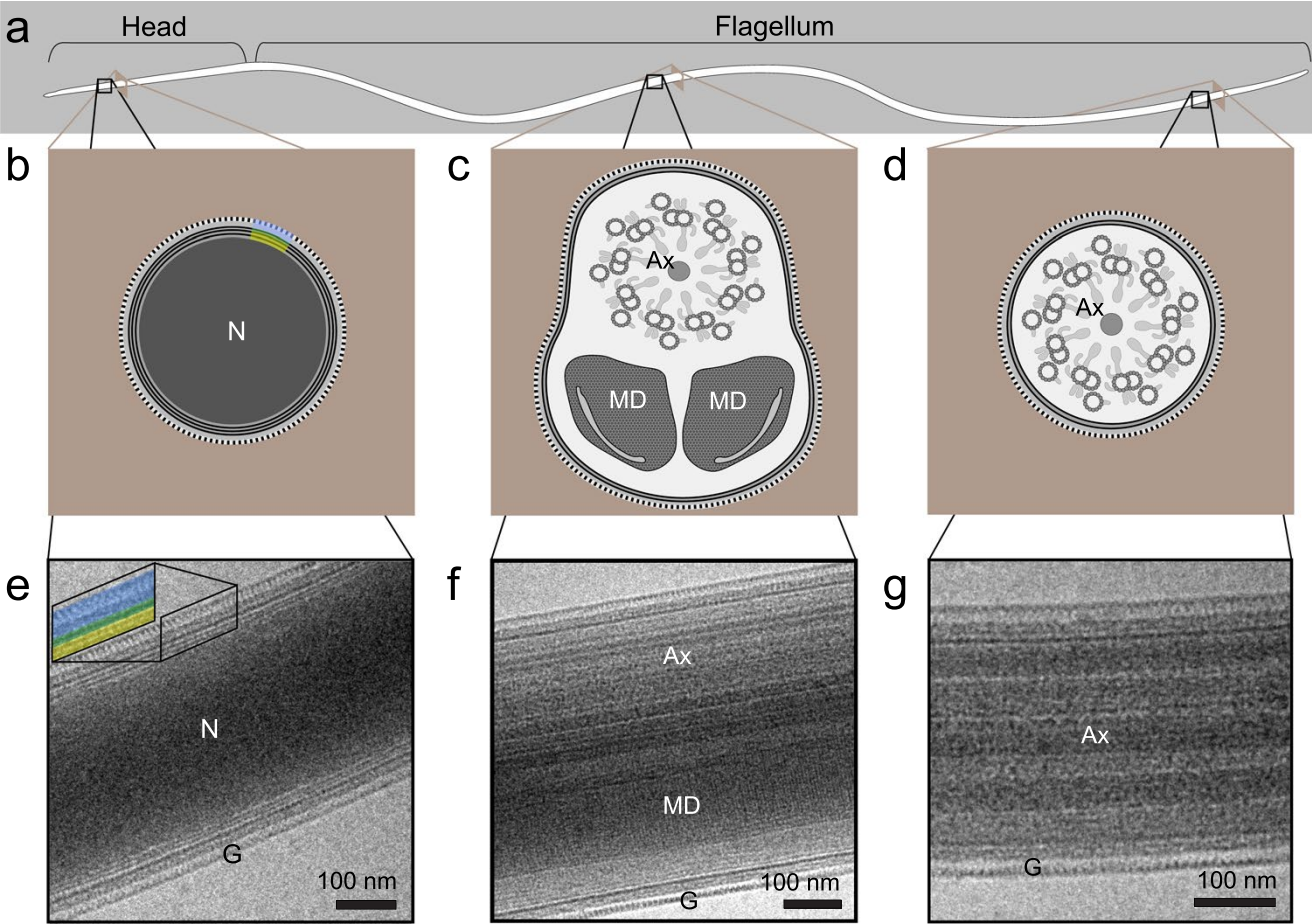
Mosquito sperm morphology overview. (**a**) Diagram of full sperm length based on light microscopy (~250 µm long; width of sperm not to scale). Sperm heads in motile sperm can be identified by their rigidity (in comparison to the motile flagellum), but otherwise look very similar to the flagellum. (**b**–**d**) Diagrams of cross sections of sperm nucleus (**b**), anterior flagellum (**c**), and posterior flagellum (**d**) are based on previous electron micrographs of sperm from *Ae. aegypti*^20^, *Culex quinquefasciatus*^18^, and *Toxorhynchites brevipalpis*^19^. (**e**–**g**) Cryo-transmission electron micrographs in lateral view (this study) of sperm nucleus (**e**), anterior flagellum (**f**), and posterior flagellum (**g**). To identify surface structure, part of the surface in b and e (inset) are color coded to indicate the nuclear envelope (yellow), plasma membrane (green), and glycocalyx (blue). Ax, axoneme; G, glycocalyx; MD, mitochondrial derivative; N, nucleus.

**Figure 2:**
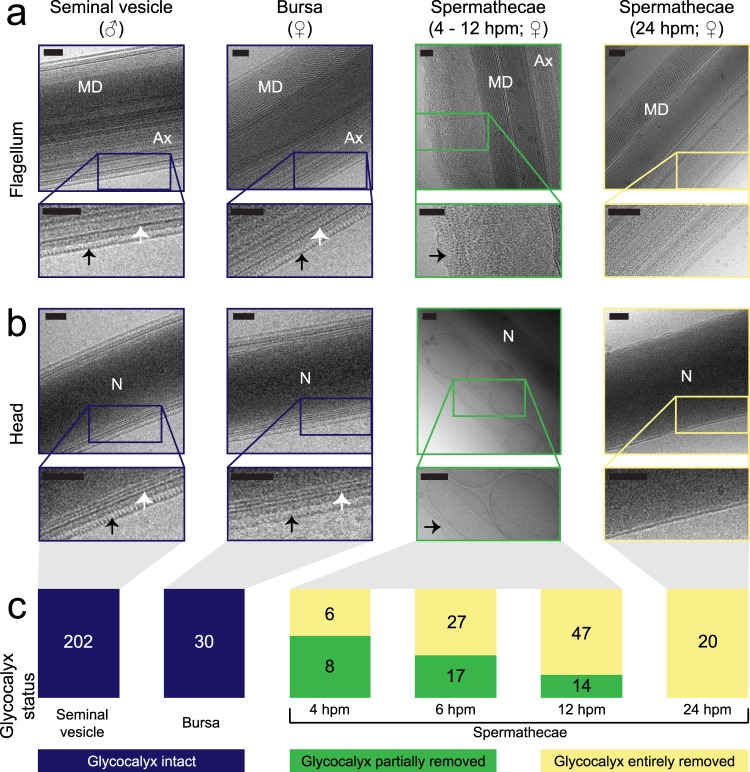
Glycocalyx presence on sperm from the male seminal vesicles and at different times post-mating in the female reproductive tract. (**a**,**b**) Lateral view of sperm flagella (**a**) and heads (**b**) from seminal vesicles (navy panels), bursa (navy panels), spermathecae at 4–12 hpm (green panels), and spermathecae at 24 hpm (yellow panels). Insets show glycocalyx morphology and demonstrate intact glycocalyx on sperm from the seminal vesicles and bursa, glycocalyx dissociating from sperm in the spermathecae at 4–12 hpm, and lack of a glycocalyx at 24 hpm. Arrows indicate glycocalyx. (**c**) Proportion of sperm with glycocalyx intact (navy), glycocalyx entirely removed (yellow), or with intermediate state of removal (green) at different times before and after mating. Split bars represent the proportion of total imaged sperm with given phenotype, and numbers in bars indicate number of sperm imaged with given phenotype. n = 11 males for sperm from the seminal vesicles, 3 females for bursa, 4 hpm, and 6 hpm, and 4 females for 12 hpm and 24 hpm. Ax, axoneme; MD, mitochondrial derivative; N, nucleus; white arrows, plasma membrane; black arrows, glycocalyx. All scale bars: 100 nm.

To understand baseline sperm morphology, we first dissected sperm from the paired seminal vesicles of sexually mature male *Ae. aegypti* mosquitoes into saline. Sperm in this organ have completely developed, but have not yet mixed with seminal fluid contributions of the accessory glands. Because of these cells’ extreme length-to-width ratio^20,21^, we show representative sections of different anatomical features, along with diagrams of their cross-sectional anatomy (Fig. 1). In general, the ultrastructure of *Ae. aegypti* sperm at this stage is similar to that described in other mosquito genera^18,19^. Mature spermatozoa were about 250 nm wide at each end, and 750 nm wide at their widest point, consistent with a previous study^20^. The needle-like, 30 µm-long head (Fig. 1a) is identifiable by its electron-dense nucleus with a homogeneous appearance, owing to its composition of tightly packed chromatin (Fig. 1b,e). The flagellum is composed of two mitochondrial derivatives that run most of the length of the flagellum, and an axoneme that consists of microtubular rings and molecular machinery that power motility and extend nearly the full length of the flagellum (Fig. 1c,d,f,g). The mitochondrial derivatives are identifiable by their paracrystalline protein structure^22^ (best seen in Fig. 2a, yellow panel; Supplementary Fig. 2d), and the axoneme appears as striations running parallel to the length of the sperm (Fig. 1c,d,f,g).

Mature mosquito sperm, like the sperm of many insects^23^, have a plasma membrane that is covered by a thick glycoprotein coat, the glycocalyx^18–20^. In *Ae. aegypti*, this coat is approximately 35 nm-thick (Figs. 1e–g, 2b, S1a–c). This layer is suggested in other mosquitoes to be deposited by secretory cells in the testes and vasa deferentia after sperm have undergone individualization^19^. The glycocalyx appears to have three strata: the innermost and outermost being electron dense with an electron translucent layer separating the two (Fig. 1e). Whereas micrographs of the glycocalyx in other mosquito sperm depict a homogeneous layer^18,19^, our stain-free cryo-TEM approach revealed a striking, repeating structure to the glycocalyx (Supplementary Fig. S1c–e), similar to that described in sperm from the grasshopper *Pezotettix giornae*^23^. In *Cx. quinquefasciatus* sperm, the glycocalyx is proposed to be composed of carbohydrates based on labeling with various lectin-gold complexes^18^, but the specific residues that comprise it remain undescribed.

We also note that sperm heads often had vesicles nestled among the condensed chromatin (Supplementary Fig. S1a,b). Because several membranes (i.e., the plasma membrane, two membranes of the nuclear envelope, and the vesicles’ one membrane) converge in a small space, it is difficult to discern from our micrographs whether these vesicles are situated inside or outside the nuclear envelope. These vesicles were ovate, with major axes of 275 ± 80 nm and minor axes of 163 ± 40 nm (mean ± SD; *n* = 26 vesicles). Their contents are more electron-dense than the surrounding cytoplasm. In another mosquito, *Cx. tigripes*^24^, similar structures have been suggested to originate from leftover membrane as the nucleus is compressed during sperm individualization. Nonetheless, in light of the sperm/vesicle interactions described in *Drosophila melanogaster*^25^ and mice^26^, it is possible that vesicles in mosquito sperm have a more nuanced purpose than simply being a byproduct of spermiogenesis.

### Sperm shed their glycocalyx within 24 h of storage in the female’s spermathecae

Two previous studies of sperm from mosquitoes in other genera suggest that mosquito sperm lose their glycocalyx after mating^18,19^. However, whether this modification occurs in *Ae. aegypti*, the timing with which it occurs, whether it occurs in all sperm or just a few, and its function remain uncertain.

Females are able to store sperm from one insemination for their entire life in spermathecae—a set of three rigid capsules that protect, maintain, and nourish sperm (reviewed in^12,27^). To compare sperm from the male’s seminal vesicles to those in the female’s spermathecae, we prepared sperm from the female at 24 h post-mating (hpm) by gently opening the medial spermatheca in saline using a minutien pin. Individual sperm were allowed to leave the spermathecae before flash-freezing them in liquid nitrogen for cryo-TEM imaging. Whereas all sperm from the male’s seminal vesicles had an intact glycocalyx (Fig. 2a,b, navy panel), this layer was absent from the whole length of all sperm harvested from the female’s spermathecae at 24 hpm (Fig. 2a,b, yellow panel). To determine whether the glycocalyx was removed as part of the sperm’s activation to motility during insemination, we next imaged sperm 3 min after insemination, when they are located in the bursa. In this organ, sperm are bathed in seminal fluid and bursal secretions and are activated to rapid motility^12^. At this point, sperm surface structure was indistinguishable from that of pre-insemination sperm from the male, with fully intact glycocalyces (Fig. 2a,b, navy panel). Finally, we examined sperm from the spermathecae at 4, 6, and 12 hpm. At these time points, an increasing number of sperm from the spermathecae did not have a glycocalyx (Fig. 2c). By contrast, those with evidence of a glycocalyx displayed an intermediate state of removal, with parts of the glycocalyx appearing to have “peeled” off of the sperm (Fig. 2a, green panel; Supplementary Fig. S2d,e), or the glycocalyx sliding off of the head intact (Fig. 2b, green panel; Supplementary Fig. S2). The repeating structure of the glycocalyx allowed for its identification during and after dissociation from sperm (Supplementary Fig. S1d). We note that the number of sperm imaged from these time points was limited by the number that left the spermathecae (see below). If the presence of the glycocalyx in some way impedes sperm motility and detachment from the sperm mass during our sample preparation, then the proportion of sperm within the spermathecae whose glycocalyx is fully removed may be lower than the subset we were able to image.

### Sperm are dormant for several hours at the same time the glycocalyx is being removed

**Figure 3:**
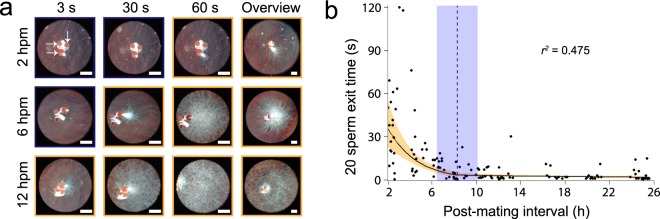
The timing with which sperm leave spermathecae cracked at different times post-mating. (**a**) Spermathecae (indicated by arrows in top left image) from representative females cracked in saline at 2, 6, and 12 hpm, and imaged at 3, 30, and 60 s post-crack. Overview taken after 120 s post-crack. Boxes in yellow indicate samples in which 20 sperm have begun to exit spermathecae. Scale bars 200 µm. For examples of motility at different times post-mating, see Supplementary Video S1. (**b**) Twenty sperm exit time of females’ spermathecae dissected between 2 and 26 hpm (*n* = 130). Solid line and accompanying orange ribbon indicate segmented linear regression with one breakpoint and 95% confidence interval; dashed line and blue ribbon indicate breakpoint of segmented linear regression and 95% confidence interval. *r*^2^ is the adjusted coefficient of determination for all data points. All calculations were performed on log-transformed data but are plotted in untransformed coordinates.

*Aedes aegypti* spermatozoa display rapid motility while they are being stored^12^ as well as within the spermathecae^28,29^. However, while harvesting sperm from the spermathecae for ultrastructural imaging, we noticed that sperm (particularly those stored for time periods less than 4 hpm) were slow to exit the spermathecae shortly after mating. To test whether sperm exhibit altered motility after storage and during glycocalyx removal, we assayed sperm activity by gently cracking the chitinous, rigid covering of the spermathecae of females at different post-mating intervals and recording the emerging sperm (Supplementary Video S1). Shortly after mating, sperm were sluggish and slow to emerge from the cracked spermathecae; they often only partially exited the spermathecae and did not escape into a free-swimming form. Those that dissociated from the sperm bundle showed compromised motility and weak swimming activity, with few traveling far from the spermathecae. However, with increasing time post-mating, sperm became more active, and upon cracking, escaped the spermathecal capsules faster (Fig. 3a). As a proxy for overall activity within the sperm mass, we calculated the time required for 20 sperm heads to emerge from the ruptured medial spermatheca.

Twenty sperm exit time initially decreased with increasing time post-mating (segmented linear regression; *df* = 55, *t* = 5.89, *p* < 0.001; Fig. 3b; Supplementary Table S1) and reached a minimum at 8.3 ± 1.9 hpm (breakpoint ± 95% CI), after which time no significant change in 20 sperm exit time was detected (*t* = 1.78, *df* = 71, *p* = 0.08; Fig. 3b; Supplementary Table S1). Variation in this metric, particularly at early time points, could be partially explained by the fact that females may receive between 400 and 4000 sperm when they are inseminated^30^.

### Oviposition and fertility plateau within 24 h of insemination

Given this timeline for sperm modification, we investigated whether other reproductive processes follow a similar timeline. To test how soon females lay eggs after mating, we blood fed virgin females, waited for them to produce eggs, and mated females at staggered intervals for 24 h. We then gave all females a 2 h oviposition period, in which they were given a moist substrate on which they could lay eggs. Finally, we attempted to hatch all eggs that females laid to assess whether females were fertile.

**Figure 4:**
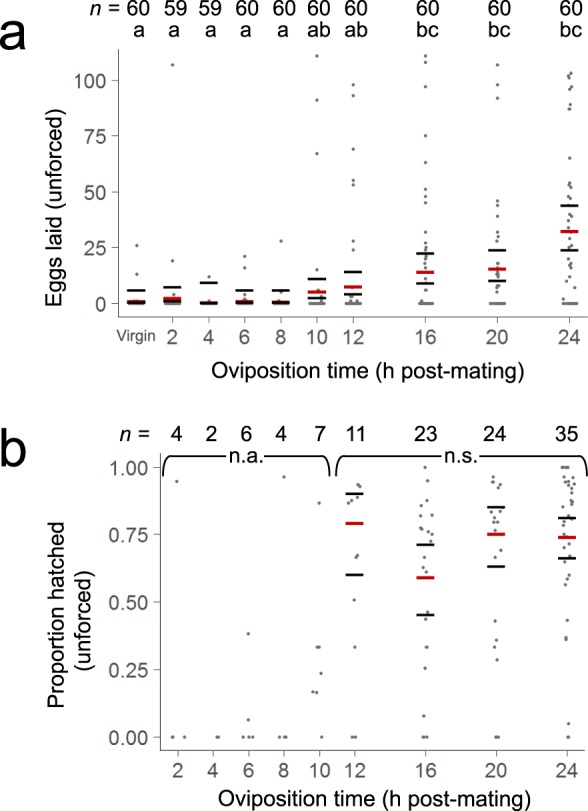
Eggs laid (**a**) and proportion of eggs that hatched (**b**) of females laying eggs of their own volition. Each plot includes females at nine different post-mating intervals; egg count plots also include virgin controls. Each dot represents one female. Times that have at least one letter in common indicate that there were no significant differences between time points (generalized linear models with Bonferroni-corrected pairwise comparisons; see text for model structure). Black lines represent 95% confidence interval, and red lines represent estimated marginal means. *n*, sample sizes; n.s., no pairwise comparisons were significantly different; n.a., groups were not included in pairwise comparisons due to low sample size.

Post-mating interval strongly influenced oviposition. Oviposition prior to 12 hpm was sporadic and did not differ significantly from oviposition by virgin females. However, a stark increase in oviposition was observed after this time (generalized linear model (GLM); *df* = 9,588, *F* = 15.05, *p* < 0.001; Supplementary Table S2; Fig. 4a). From 12–24 hpm, post-mating interval did not significantly affect hatch rate (GLM; *df* = 3,88, *F* = 1.39, *p* = 0.25; Supplementary Table S2; Fig. 4b). Most females (88%) that laid eggs at or after 10 hpm produced viable larvae (*n* = 88 of 100), whereas prior to 10 hpm, only 25% of mated, egg-laying females (*n* = 4 of 16) produced eggs that hatched (Fig. 4b).

Finally, we aimed to quantify how soon after mating females gain the capacity to fertilize eggs. Most females did not lay eggs until after 10 hpm, but because oviposition in *Ae. aegypti* is triggered by hormones received during mating (and not necessarily fertility)^31,32^, we reasoned that some females may become fertile sooner than they are stimulated to lay eggs. To characterize when females become fertile after mating, we developed an assay to force females to lay their fully developed eggs prior to 10 hpm using death stress (Supplementary Discussion). Fertility (as measured by hatch rate) of females laying eggs in this manner increased sigmoidally from 4 hpm to 16 hpm, with the sharpest increase from 8 hpm to 12 hpm (Supplementary Fig. S3; Supplementary Table S2). Overall, females that were forced to lay their eggs via death stress had slightly lower hatch rates than females that laid eggs of their own volition (GLM; *df *= 1,244, *F *= 20.90, *p* < 0.001; Supplementary Table S2).

## Discussion

**Figure 5:**
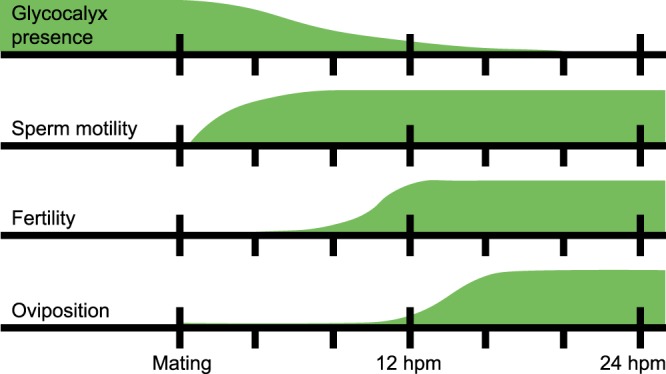
Timelines demonstrating the onset of facets of the post-mating response tested in this study. Green bars represent the proportion of sperm with a glycocalyx, degree of sperm motility in the spermathecae, proportion of eggs that are fertilized, and the number of females laying eggs before mating and for 24 h post-mating. Highest points on each bar are scaled to the maximum proportion (glycocalyx presence, fertility, oviposition) or value (sperm motility) observed in this study. Curves are based on values measured in this study and are smoothed to simplify their interpretation.

Herein, we describe the timing of four previously undescribed events in *Ae. aegypti* reproductive physiology: (a) the loss of a sperm glycocalyx after insemination, (b) a period of sperm inactivity that coincides with the removal of the glycocalyx, and (c) the onset of female fertility, and (d) oviposition behavior, both of which occur at the same time or shortly after sperm are modified (Fig. 5). We discuss these findings in the context of challenges sperm may face in the female reproductive tract and reaching their ultimate objective to fertilize eggs.

Sperm in the male’s seminal vesicles are mostly inactive. However, during insemination, they are deposited in the female’s bursa (a temporary holding sac), where they almost immediately become rapidly motile^12^. The glycocalyx may function to protect sperm prior to storage (reviewed in^13^). While no threats to mosquito sperm have been empirically demonstrated during their ~5 min stint in the bursa, evidence from mosquitoes and examples from other systems suggest that sperm may experience hazards shortly after insemination. For example, mosquito seminal fluid is loaded with proteases and peptidases^33–36^. While some proteases likely function to activate sperm motility^37^, it is possible that the catabolic cocktail in seminal fluid would be detrimental to an unprotected cell. In *Drosophila pseudoobscura*, a proportion of sperm are killed within 30 min of insemination, likely due to female reproductive tract secretions^38^. In mammals, sperm are equipped with a thick outer glycocalyx that is covered in glycoproteins and sialic acid residues that mask and defend the cell from the female immune system^39,40^. The *Ae. aegypti* glycocalyx resembles that of orthopteran sperm^23^, and a similar loss of the glycocalyx has been described in members of this order^41,42^. Understanding the molecular composition of the glycocalyx in mosquitoes and the hazards sperm face after insemination may illuminate the function of the glycocalyx.

Once in the spermathecae, sperm activity drastically slows after a period of extreme motility in the bursa, in agreement with previous observations in *Ae. aegypti*^29^ and *An. gambiae*^43^. While seminal fluid is assumed to nourish sperm while they are in the bursa, the spermathecae and their associated secretory cells likely take over this role after sperm are stored (reviewed in^44^). We propose that sperm’s dormant period may occur due to a lag in the time it takes for spermathecal secretory cells to begin providing sustenance to sperm. It is also possible that sperm initially form a tangled mass in storage, but disentangle themselves as they lose their glycocalyx and become motile. In support of this hypothesis, we note that sperm at 2 hpm in Supplementary Video S1 appear moderately motile but disorganized and unable to escape the sperm mass, whereas at 6 hpm, sperm heads spill out as if they were oriented in the same plane and direction, and sperm are able to escape the sperm mass with ease. A similar progression of events has been observed in the fungus gnat *Sciara coprophila*^45^.

Sperm’s move to the spermathecae may also involve being nourished by a new suite of metabolic pathways. In honeybees, seminal fluid and spermathecal fluid have vastly different metabolic networks^46^, and the abundance of certain metabolic enzymes in honeybee sperm differs between freshly ejaculated and stored sperm^47^. Could the loss of the glycocalyx (and perhaps the plasma membrane) play a role in this transition? It is possible that sperm’s original protective coat enables a degree of intracellular homeostasis that is required for sperm to produce their own energy, and that once sperm are in the nutritive spermathecae, their energetic needs are provided exogenously. Spermathecal secretory cells likely also provide antioxidant services, as they do in *An. gambiae*^48^, and such a thick outer layer may impede the provisioning of peroxidases and other protective enzymes to sperm. A deeper understanding of mosquito sperm metabolism, sustenance by the spermathecae, and sperm motility may explain why the glycocalyx is removed.

While mosquitoes can be forced to oviposit shortly after copulation, little natural oviposition occurs before 10 hpm, but drastically increases after 12 hpm. What stimulates natural oviposition at this time period? One possibility is that once females are capable of fertilizing eggs, an as yet undescribed feedback mechanism signals readiness to fertilize eggs and initiates oviposition. However, oviposition is likely more tightly linked to a seminal fluid component(s) rather than the actual ability to fertilize eggs. Intrathoracic injection of virgin females with a homogenate of male accessory glands (the primary producer of seminal fluid) induces them to lay eggs^31,32^, despite never receiving sperm. A similar response to the receipt of seminal fluid molecules has been described for insects in several other taxa (reviewed in^49^). In addition, oviposition in our experiments did not always result in viable offspring, even when a female had been naturally inseminated. Therefore, a transferred seminal fluid molecule likely plays the major role in stimulating oviposition, and our experiments suggest that its timing is tightly coordinated with when females become fertile. It remains a possibility that fertility plays some role in inducing oviposition, although if it does, its impact is likely minor.

While long term female monogamy is well-established in *Ae. aegypti*^5,6^, the reason that females do not regain receptivity remains unclear. The timing of the events in this study may help to explain the *Ae. aegypti* mating system. We have previously reported that some *Ae. aegypti* females mate more than once, but this most often occurs prior to 2 hpm, and only rarely occurs after 16 hpm^6^. It is conceivable that once sperm are stored, shed their glycocalyx, and become motile, conditions in the spermathecae are adjusted to sustain and protect sperm rather than transform them, and females can no longer accept and process a second cohort of sperm. Females may also shift their reproductive agenda to focus on taking blood meals and laying eggs once they are fertile. Manipulative experiments using forced copulation^29,50^, artificial insemination^51–53^, or genetic manipulations of female behavioral pathways^54^ may shed light on the proximate and ultimate reasons for long-term female monogamy in *Ae. aegypti*.

Our descriptions of post-copulatory, pre-zygotic sperm modification, fertility, and oviposition offer valuable insight into how mosquitoes reproduce. Knowing how and when sperm undergo changes may help to answer key questions regarding mosquito reproductive physiology, including how sperm motility is directed; how they are stored; which metabolic pathways sustain them; how they interact with seminal fluid and the female reproductive tract; and whether certain modifications are prerequisites for fertilization. Identifying the molecular and cellular underpinnings of such processes may lead to the development of methods to manipulate mosquito reproduction for the purpose of controlling mosquito-borne diseases. Finally, understanding how sperm are modified to cope with potential hazards, interact with the female, and fertilize eggs is important for understanding the biology of many internally fertilizing organisms, including livestock and humans. Our description of *Ae. aegypti* reproduction may provide new perspectives for investigators of reproductive physiology in diverse organisms.

## Methods

### Rearing

All mosquitoes were *Aedes aegypti* Thai strain and reared as described in^6^.

### Plunge-freezing vitrification

Using low retention pipette tips, 3 μL of sperm suspended in phosphate buffered saline designed to be isotonic to mosquito hemolymph (as described in^34^; hereafter “saline”) were pipetted onto holey carbon-coated 200 mesh copper grids (Quantifoil Micro Tools, Jena, Germany; hereafter “TEM grids”). R2/1 Quantifoil grids with hole sizes of ~2 μm were chosen to increase cell coverage. The TEM grids were blotted from the reverse side and immediately plunged into a liquid ethane/propane mixture at liquid nitrogen temperature using a custom-built vitrification device (MPI, Martinsried, Germany). The plunge-frozen TEM grids were stored in sealed cryo-boxes in liquid nitrogen until used.

All sperm imaged were from males or females aged 5–8 d post eclosion (dpe) and were dissected in saline. To collect mature, pre-insemination sperm, males’ seminal vesicles were isolated in saline, torn open at the anterior end, and gently stroked with a minutien pin to release sperm into solution. The dense sperm cloud was immediately aspirated and transferred to a TEM grid. Sperm from the bursae were collected by observing a male mating to a virgin female, dissecting the intact bursa from the female 3 min after copulation, and rupturing the bursa to allow sperm to swim into solution prior to pipetting them onto the TEM grid. Spermathecal sperm were collected by dissecting spermathecae from mated females at 4, 6, 12, and 24 h post-mating. Spermathecae were cracked using a minutien pin, given 10–15 s to allow sperm to swim out, and emerging sperm were carefully pipetted onto the TEM grid. To ensure that this 10–15 s delay was not the cause of altered sperm ultrastructure, we conducted a control experiment in which mature sperm from the seminal vesicles were prepared with this delay (n = 2 males), and we observed no morphological changes from seminal vesicle sperm prepared without delay (data not shown).

### Cryo-transmission electron microscopy

Cryo-TEM was performed on a Titan Themis (Thermo Fisher Scientific, Waltham, MA) operated at 300 kV in energy-filtered mode equipped with a field-emission gun, and either a 2048 × 2048 Ceta 16 M (Thermo Fisher Scientific, Waltham, MA), or a 3838 × 3710 pixel Gatan K2 Summit direct detector camera (Gatan, Pleasanton, CA) operating in counted, dose-fractionated modes. Images were collected at defocii of -1 μm and -3 μm. Images were binned by 2, resulting in pixel sizes of 0.51–1.09 nm. TEM grids were initially scanned at low magnification to locate sperm, and any sperm that were located were examined at high resolution, provided they were located over a hole on the TEM grid and not embedded in crystalline ice. In post-mating samples, care was taken to image as much of each sperm’s length as possible to understand how surface morphology changes over the entire cell.

### Motility assays

Females 3–14 dpe were allowed to mate with males for an hour at a 1:1 ratio. After this mating period, males were removed and females were maintained in an environmental chamber set at 71.9 ± 9.5% RH and 29 ± 1.0 °C until assayed for motility. Females were dissected in saline between 2 and 26 hpm. Each female’s spermathecal triplet was removed and transferred to 60 µL of fresh saline. A glass coverslip was gently placed over the spermathecae, and the uncracked spermathecae were placed on a compound scope with 200x total magnification and dark-field illumination. The largest (medial) spermatheca was cracked with a coverslip by slowly wicking saline from the side of the coverslip with a Kimwipe. Immediately after cracking, the Kimwipe was removed to leave sufficient saline for sperm to move and exit the spermathecae. Videos of the spermathecae and the sperm they contained were recorded from the time of cracking for 2 min. Any spermathecae that cracked before the video was recorded or that had tissue (oviduct or spermathecal ducts) that covered the location of the crack were removed from analysis. As an estimation of overall sperm motility inside the spermathecae, the time taken for 20 sperm heads to emerge from the medial spermatheca was recorded.

### Unforced oviposition

Virgin females 2–4 dpe were offered a blood meal from ECD’s arm after having been starved of sugar for 24 h. Females that did not feed were given a second opportunity to feed later that day. Females that still did not feed were discarded. After feeding, females were given 10% sucrose *ad libitum* and held in an environmental chamber as described above and allowed to develop eggs. At 6 d post-feeding, 35 females were combined with 35 males and allowed to mate for 2 h, after which males were removed. Over the next 24 h, eight more groups of females were similarly mated at 2 or 4 h intervals prior to oviposition. The mating status of 177 females was verified by checking their spermathecae for sperm, indicating that 92% of females successfully mated. At 7 d post-feeding, all females were given 2 h to oviposit at the same time, such that the nine groups of females had 2, 4, 6, 8, 10, 12, 16, 20, or 24 h between their mating period and oviposition period. A virgin control group was also given the same opportunity to oviposit. Oviposition took place in a 454 mL cup with a 30 mL oviposition cup lined with wet paper towel as oviposition substrate and filled with 10 mL deionized (DI) water. After 2 h, females were removed and the eggs they laid were counted. After 1 d, water was removed from each cup, paper towels were allowed to dry until they were slightly moist, and egg sheets were placed in a container with wet paper towel to maintain 100% relative humidity. After 3 d, eggs were submerged with DI water and a pinch of ground fish food (Hikari, Himeji, Japan), subjected to vacuum pressure for 30 min, and allowed 5 d more to hatch. Finally, larvae were counted. Each post-mating interval included 30 females, and this entire experiment was conducted twice with different cohorts of mosquitoes.

### Death stress oviposition

Gravid virgins were prepared as described above for unforced oviposition experiments. Females were mated for 30 min at the same intervals and sex ratios as unforced females. In this experiment, rather than providing oviposition cups to females, their heads were removed with forceps at the appropriate post-mating interval, stimulating death stress oviposition. Decapitated carcasses were placed individually on top of a wet filter paper disk in the well of a 96-well plate. Each well was covered with masking tape and the plate was placed in a plastic bag with a wet paper towel. Females were allowed to death stress oviposit for 1 h, after which they were removed from the wells and the masking tape was replaced. All females’ spermathecae were then dissected to verify that they had mated, and females that had no sperm were pooled into a virgin control treatment. To avoid mold growth, excess water was removed from each well 1 d after oviposition. Eggs were allowed 3 d to embryonate, after which they were counted and scored as either viable (convex, fully melanized, and normally shaped) or inviable (deflated, not melanized, or irregularly shaped). At times, females laid many eggs on top of each other, preventing their exact enumeration without damaging the eggs. In such cases, we estimated the total number of eggs by counting those that were visible and estimating how many were not visible based on the depth of the pile of eggs. Wells in which mold grew were recorded. Occasionally, some eggs exhibited a phenotype in which the whole chorion was melanized, but horizontal stripes that lacked tubercles gave portions of the egg a glassy black appearance. We also recorded these wells. After counting and characterizing the eggs in each well, wells were flooded with 200 µL DI water and returned to the environmental chamber for 7 d. After 7 d, eggs were subjected to vacuum pressure for 30 min to stimulate hatching, after which the total number of hatched larvae in each well was counted. This entire experiment was conducted twice with different cohorts of mosquitoes. Each replicate initially included 30 or 32 females per treatment.

### Statistical analysis

Segmented linear regression to analyze motility was conducted in R (v. 3.4.2, Vienna, Austria). Models to analyze oviposition were created using SPSS (IBM SPSS Statistics for Windows, v. 24.0, Armonk, NY).

#### Motility

Changes in sperm motility were assessed using linear regression, with log-transformed twenty sperm exit times as the dependent variable and post-mating interval (calculated by the beginning of grouped mating periods to the time of dissection) as the independent variable. The time at which we stopped recording was used for the twenty sperm exit time of two females (out of 130) whose sperm did not leave the spermathecae. Log-transformed data were both normally distributed and fit the assumption of homoscedasticity. Because our initial simple linear regression indicated that twenty sperm exit time reached a minimum near 8 hpm, we conducted a segmented linear regression (package: “segmented”^55^) to quantitatively assess where sperm motility plateaued. Whether the slope of each segment differed significantly from zero was tested using two-sided one sample *t* tests.

#### Oviposition

All oviposition data was analyzed using generalized linear models. Models assessing the number of eggs laid by each female had a Poisson distribution with a log link function, and all models assessing egg viability or hatch rate had a binomial distribution with a logit link. Because all data were overdispersed, the Pearson Chi-square was used as a scale parameter in each model. Models began with a fully factorial design, followed by iterative removal of the least significant term until all terms were significant (*p* < 0.05). Final model terms are included in Supplementary Table S2. To compare response variables at different post-mating intervals, post-hoc pairwise testing was conducted using two-sided least significant difference comparisons with a Bonferroni correction (Fig. 4).

#### Unforced oviposition

To assess when females are stimulated to lay eggs, post-mating interval and replicate were included as a factor, and eggs laid as a response variable. All females were included in the analysis (*n *= 59–60 per post-mating interval). To test whether post-mating interval affects fertility, each egg’s hatch status was used as the response variable, and post-mating interval and replicate were included as factors. Because no more than seven females laid eggs prior to 12 hpm (Fig. 4b), only females at 12, 16, 20, and 24 hpm were included in pairwise comparisons.

#### Death stress oviposition

All wells in which any mold grew were removed from analysis (*n* = 128). For analysis of the total number of eggs laid, all remaining females were included (*n* = 418), with 40–48 females per post-mating time interval. A GLM was constructed in the same way as for unforced oviposition. For analysis of the proportion of eggs that were viable, all females that laid at least one egg were included (*n* = 367), with 37–44 females per post-mating time interval. Each egg’s viability was used as the response variable, and post-mating interval, whether wells contained striped eggs, and replicate were included as factors. For analysis of fertility, only mated females with at least one viable egg were included (*n* = 346), with 37–44 females per interval. Whether each viable egg hatched was included as a response variable; factors included post-mating interval, replicate, and whether wells had striped eggs. Several wells (9 out of 367) had slightly more larvae counted than the number of eggs that were initially counted, due to the need to occasionally estimate egg number. In these cases, the difference between larvae and viable egg counts was at most 8, and was on average 4.3. Because all of these instances were in the 16, 20, or 24 hpm time points, we kept these females in our analysis to avoid skewing data for only later time points. To ensure that these data fit our model’s structure, we forced the number of larvae hatched in these wells to the number of viable eggs that were initially counted.

#### Unforced vs. death stress oviposition

Hatch rate of egg-laying females from 12, 16, 20, and 24 hpm for both oviposition methods was compared using each viable egg’s hatch status as a response variable, and oviposition type and post-mating interval as factors. All eggs laid via unforced oviposition were assumed to be viable after several egg sheets were examined and found to have no concave or unmelanized eggs.

## Supplementary information

Supplementary information

Supplementary information

Supplementary information

## Data Availability

The authors declare that the data supporting the findings of this study are available within the paper and in Supplementary Table S3. Cryo-EM images, videos of sperm motility, and the mosquito strain used in this study are available from corresponding authors upon reasonable request.
