# Supplementary material for: Cryo-Electron Microscopy Reveals That Sperm Modification Coincides with Female Fertility in the Mosquito Aedes aegypti
Source: Sci Rep. 2019 Dec 6;9:18537. doi: 10.1038/s41598-019-54920-6 (PMC6898104; doi:10.1038/s41598-019-54920-6)
Supplement: Supplementary file 1 — Supplementary information [file 41598_2019_54920_MOESM1_ESM.docx]

CRYO-ELECTRON MICROSCOPY REVEALS THAT SPERM MODIFICATION COINCIDES WITH FEMALE FERTILITY IN THE MOSQUITO *AEDES AEGYPTI*

Jade M. Noble; Ethan C. Degner; Laura C. Harrington; Lena F. Kourkoutis

**Supplementary Material**

Supplementary Figure S1 2

Supplementary Figure S2 3

Supplementary Figure S3 4

Supplementary Video S1 (caption) 5

Supplementary Table S1 6

Supplementary Table S2 7

Supplementary Discussion: Death Stress Oviposition 8-10

Supplementary Figure S4 11

Supplementary Table S3 (caption) 12


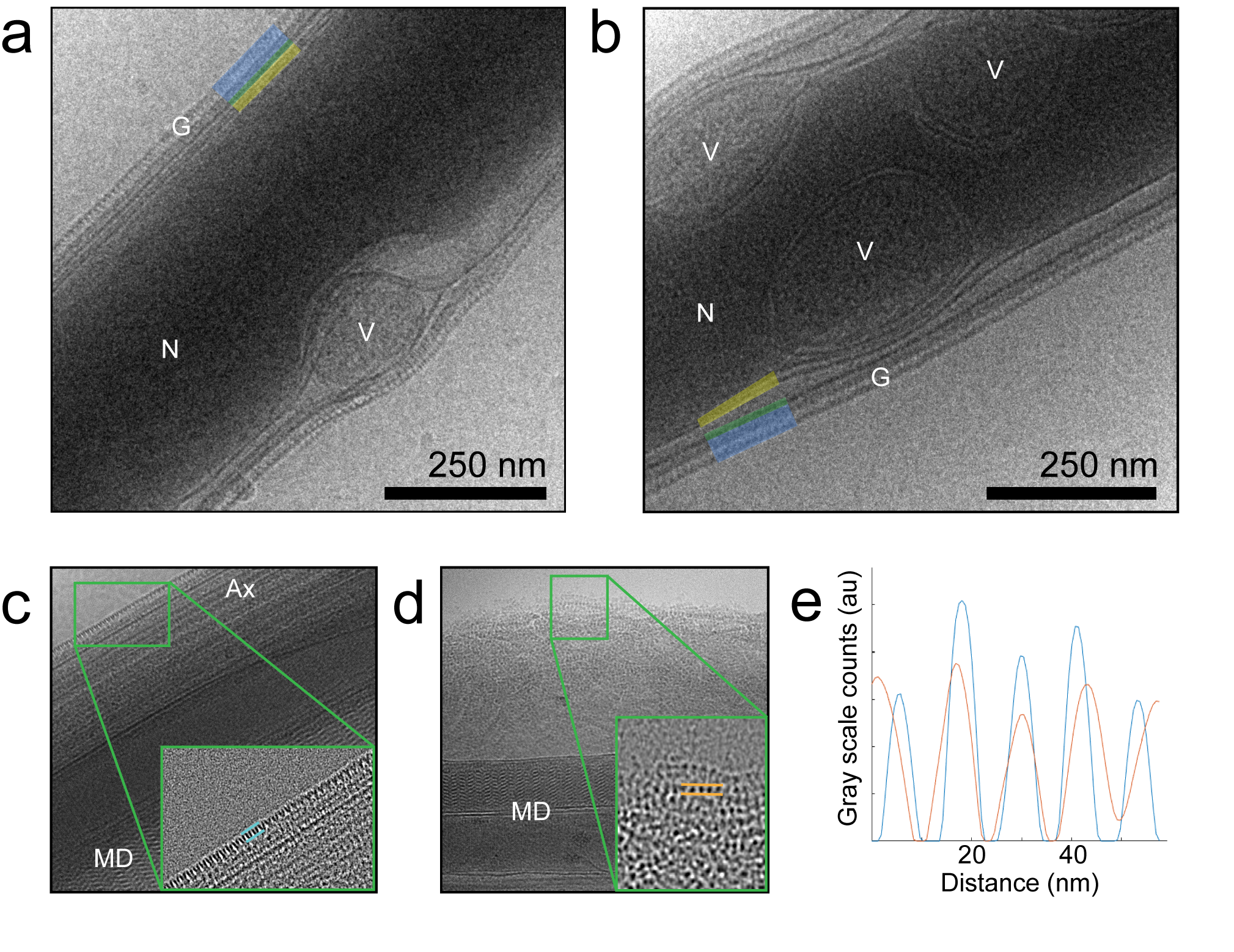
**Supplementary Figure S1.** Novel observations of *Ae. aegypti* sperm morphology. (a-b) Vesicles nestled within the sperm head. (c-d) Glycocalyx with Gaussian blur (σ = 2; large images) and bandpass filter (< 6.4 nm, > 21 nm; insets) for intact glycocalyx (c) and glycocalyx peeling off (d). (e) Pixel intensity of the segment straddled by blue (c) and orange (d) parallel lines, highlighting the repeating structure that makes up the glycocalyx and assists in identifying the glycocalyx after it has dissociated from the cell (d). Ax, axoneme; G, glycocalyx; MD, mitochondrial derivative; N, nucleus; V, vesicle; au, arbitrary units. Shading of surface layers (a-b): nuclear envelope (yellow), plasma membrane (green), and glycocalyx (blue).

**
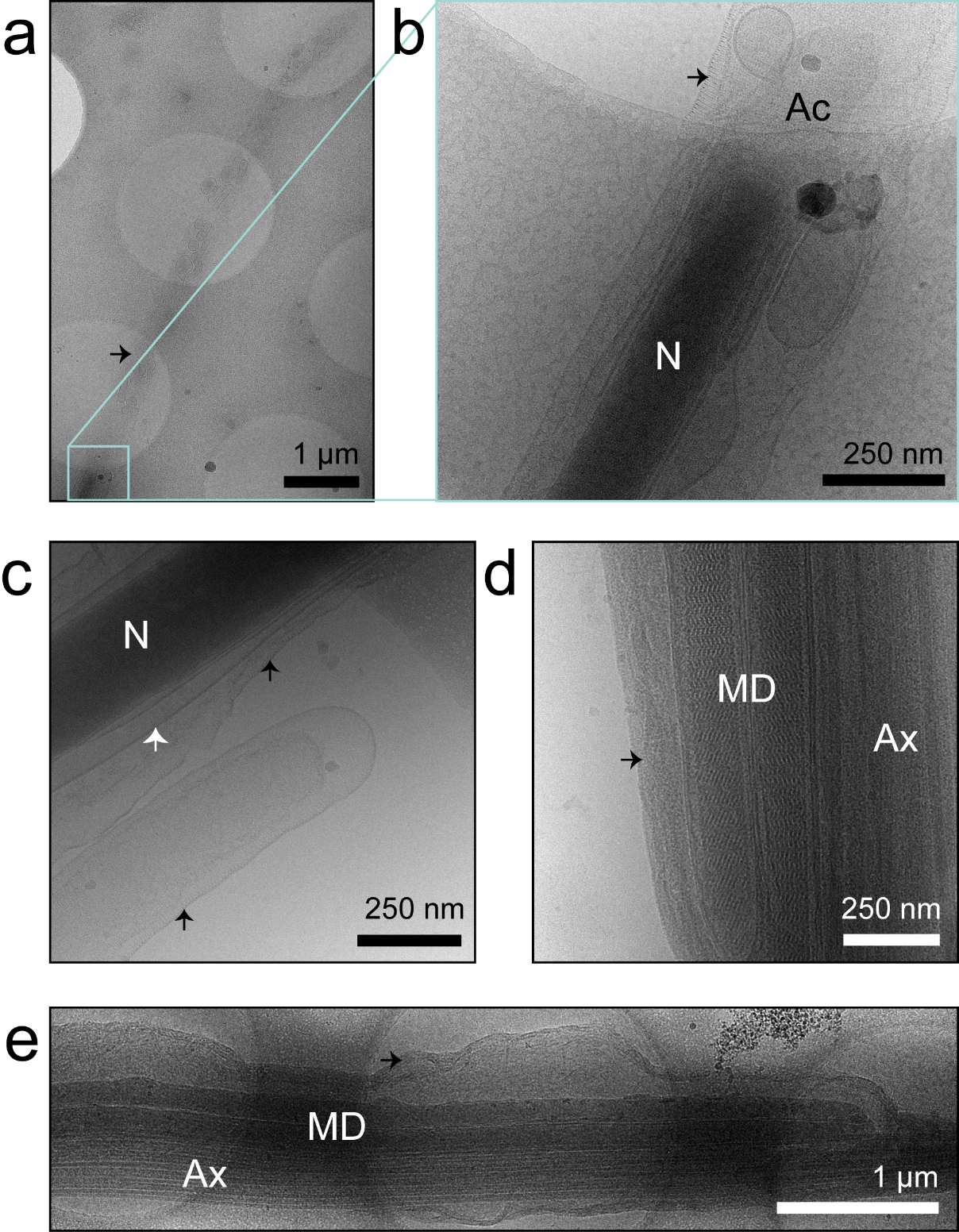
**

**Supplementary Figure S2**. Images of the glycocalyx being sloughed from the sperm. (a and b) Empty glycocalyx shell (a), with inset (b) displaying the tip of the nucleus within the dissociating glycocalyx. (c) Tip of a glycocalyx shell alongside a nucleus whose glycocalyx is dissociating from the cell. (d and e) Glycocalyx peeling off the flagellum. Note the paracrystalline structure clearly visible in the mitochondrial derivatives of (d). Black arrows indicate glycocalyx; white arrow indicates plasma membrane. Ac, acrosome; Ax = axoneme; MD = mitochondrial derivative; N = nucleus.


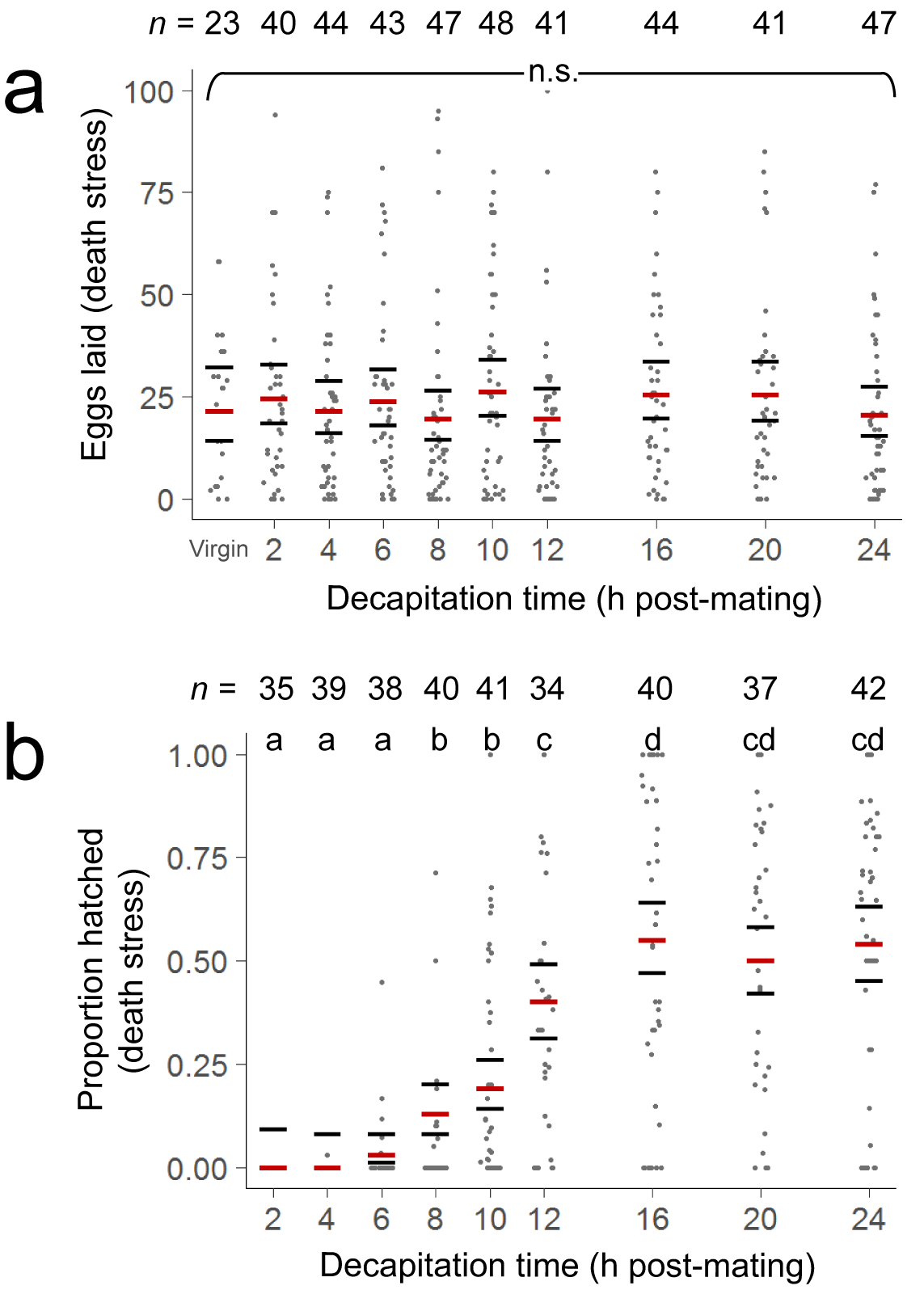


**Supplementary Figure S3**. Eggs laid (a) and proportion of eggs that hatch (b) from mated females laying eggs via death stress oviposition. Each plot includes the same females that were decapitated at nine different post-mating intervals; egg count plots also include virgin controls. Times that have at least one letter in common indicate no significant differences between time points (generalized linear models with Bonferroni-corrected pairwise comparisons; see Methods for model structure). Black lines represent 95% confidence interval, and red lines represent estimated marginal means.**Supplementary Video S1.** Sperm motility at different times post-mating. Three sets of spermathecae were taken from females at 2 hpm (left), 6 hpm (middle), and 18 hpm (right).

**Supplementary Table S1.** Parameters of segmented linear regression. Values represent log-transformed data.

| **Segment** | **Slope ± SE** | ***t*** | **95% CI** | ***n*** |
| --- | --- | --- | --- | --- |
| First | -0.380 ± 0.064 | 5.89 | -0.507 – -0.252 | 73 |
| Second | -0.035 ± 0.020 | 1.78 | -0.074 – 0.004 | 57 |

**Supplementary Table S2.** Parameters for GLMs investigating oviposition by unforced and decapitated females.

| **Oviposition experiment** | **Model response variable** | **Variables in model** | **Likelihood ratio χ2** | ***df1*** | ***df2*** | ***p*** | ***F*** |
| --- | --- | --- | --- | --- | --- | --- | --- |
| *Unforced oviposition* | Number of eggs laid |  |  |  |  |  |  |
|  |  | Intercept | 4.94 | 1 | 588 | 0.027 | 4.94 |
|  |  | Post-mating interval | 135.47 | 9 | 588 | < 0.001 | 15.05 |
|  | *Omnibus test* |  | 135.47 | 9 |  | < 0.001 |  |
|  | Number of hatched eggs (≥ 12 hpm) |  |  |  |  |  |  |
|  |  | Intercept | 52.76 | 1 | 91 | < 0.001 | 52.76 |
|  |  | Replicate | 4.23 | 1 | 91 | 0.043 | 4.23 |
|  | *Omnibus test* |  | 4.23 | 1 |  | 0.04 |  |
| *Death stress oviposition* | Number of eggs laid |  |  |  |  |  |  |
|  |  | Intercept | 1256.194 | 1 | 408 | < 0.001 | 1256.194 |
|  |  | Post-mating interval | 5.583 | 9 | 408 | 0.78 | 0.62 |
|  | *Omnibus test* |  | 5.58 | 9 |  | 0.781 |  |
|  | Number of healthy eggs |  |  |  |  |  |  |
|  |  | Intercept | 1125.57 | 1 | 357 | < 0.001 | 1125.57 |
|  |  | Post-mating interval | 21.20 | 9 | 357 | 0.012 | 2.36 |
|  | *Omnibus test* |  | 21.20 | 9 |  | 0.012 |  |
|  | Number of hatched eggs |  |  |  |  |  |  |
|  |  | Intercept | 328.15 | 1 | 337 | < 0.001 | 328.15 |
|  |  | Post-mating interval | 395.22 | 8 | 337 | < 0.001 | 49.40 |
|  | *Omnibus test* |  | 395.22 | 8 |  | < 0.001 |  |
| *Comparison of death stresss and unforced oviposition* | Number of hatched eggs (≥ 12 hpm) |  |  |  |  |  |  |
|  |  | Intercept | 48.17 | 1 | 244 | < 0.001 | 48.17 |
|  |  | Oviposition method | 20.90 | 1 | 244 | < 0.001 | 20.90 |
|  | *Omnibus test* |  | 20.90 | 1 |  | < 0.001 |  |

**Supplementary Discussion**

*Death Stress Oviposition*

Because little natural oviposition occurred prior to 10 hpm, we developed an assay using death stress oviposition to force females to lay their developed eggs. If gravid *Ae. aegypti* are given a death stressor, they lay as many eggs as they are able in rapid succession^1-3^. Fertilization occurs as eggs are being laid, and these death stressed females will also fertilize eggs if they are able^3^. Therefore, we prepared and mated gravid virgins at 2 h intervals in the same way as females in unforced oviposition experiments (main text). However, instead of providing oviposition substrate for these females, we decapitated them to force oviposition. We recorded the number of eggs they laid, the proportion of those eggs that were viable (as identified by a convex shape and full melanization), and the proportion that hatched. Females at all time points (including virgins) laid a similar number of eggs (GLM; *df* = 9,408, *F* = 0.62, *p* = 0.78; Supplementary Table 2; Fig. 4c). In addition, all time intervals had high proportions of viable eggs, ranging from 85–95%. While post-mating interval was significantly associated with the proportion of viable eggs laid by a female (GLM; *df* = 9,357, *F* = 2.36, *p* = 0.012; Supplementary Table 2), no significant difference in egg viability existed among time points after multiple comparison correction (Bonferroni-corrected pairwise comparisons, *p* > 0.05; Supplementary Fig. 3). Despite similar egg numbers and viability across all time points, the proportion that hatched drastically increased from 6 hpm to 16 hpm (GLM; *df* = 8,337, *F* = 49.4, *p* < 0.001; Supplementary Table 2), after which hatch proportions did not change significantly (Bonferroni-corrected pairwise comparisons, *p* > 0.05; Fig. 4d).

While eggs laid in response to death stress appeared morphologically identical to those laid by unforced females, it remains a possibility that certain events during ovulation or fertilization are less efficient when a female lays all of her eggs in quick succession. For example, the female accessory gland (a small, bulb-like secretory organ whose duct is adjacent to the site of fertilization^4^) is thought to produce secretions that are released during oviposition^5,6^, and death stress oviposition may force eggs to be laid prior to the replenishment of such secretions. In support of this hypothesis, overall hatch rate was higher in eggs laid from unforced females. Despite this potential inefficiency, laying and fertilizing eggs when faced with certain death is a natural response^1-3^ and likely serves to maximize the fitness of females that drown while ovipositing. Therefore, we propose that death stress oviposition is a valuable tool with which to study reproductive mechanisms, particularly shortly after insemination. Almost no females were fertile with this method through 4 hpm, but from 6–16 hpm, fertility increased drastically. Given that this change coincides with sperm losing their glycocalyx and becoming motile, a causal link between sperm modification and fertility is not inconsistent with our data. This study provides the groundwork for future investigations of the physiological prerequisites of fertilization.

**Supplementary References**

56 Decoursey, J. D. & Webster, A. P. Effect of insecticides and other substances on oviposition by *Aedes sollicitans*. *J. Econ. Entomol.* **45**, 1030-1034 (1952).

57 Wallis, R. J. & DeBishop, J. Death-stress oviposition by *Aedes canadensis*. *J. Econ. Entomol.* **50**, 112 (1957).

58 Chadee, D. D. & Ritchie, S. A. Oviposition behaviour and parity rates of *Aedes aegypti* collected in sticky traps in Trinidad, West Indies. *Acta Trop.* **116**, 212-216 (2010).

59 Degner, E. & Harrington, L. Sperm storage in a menacing mosquito. *Mol. Reprod. Dev.* **83**, 469 (2016).

60 Masci, V. L. *et al.* Reproductive biology in Anophelinae mosquitoes (Diptera, Culicidae): fine structure of the female accessory gland. *Arthropod Struct. Dev.* **44**, 378-387 (2015).

61 Rossignol, P. A., McIver, S. B. & Goldenberg, M. Accessory reproductive gland of female *Aedes aegypti*: structure and relationship to oogenesis. *Ann. Entomol. Soc. Am.* **70**, 279-281 (1977).


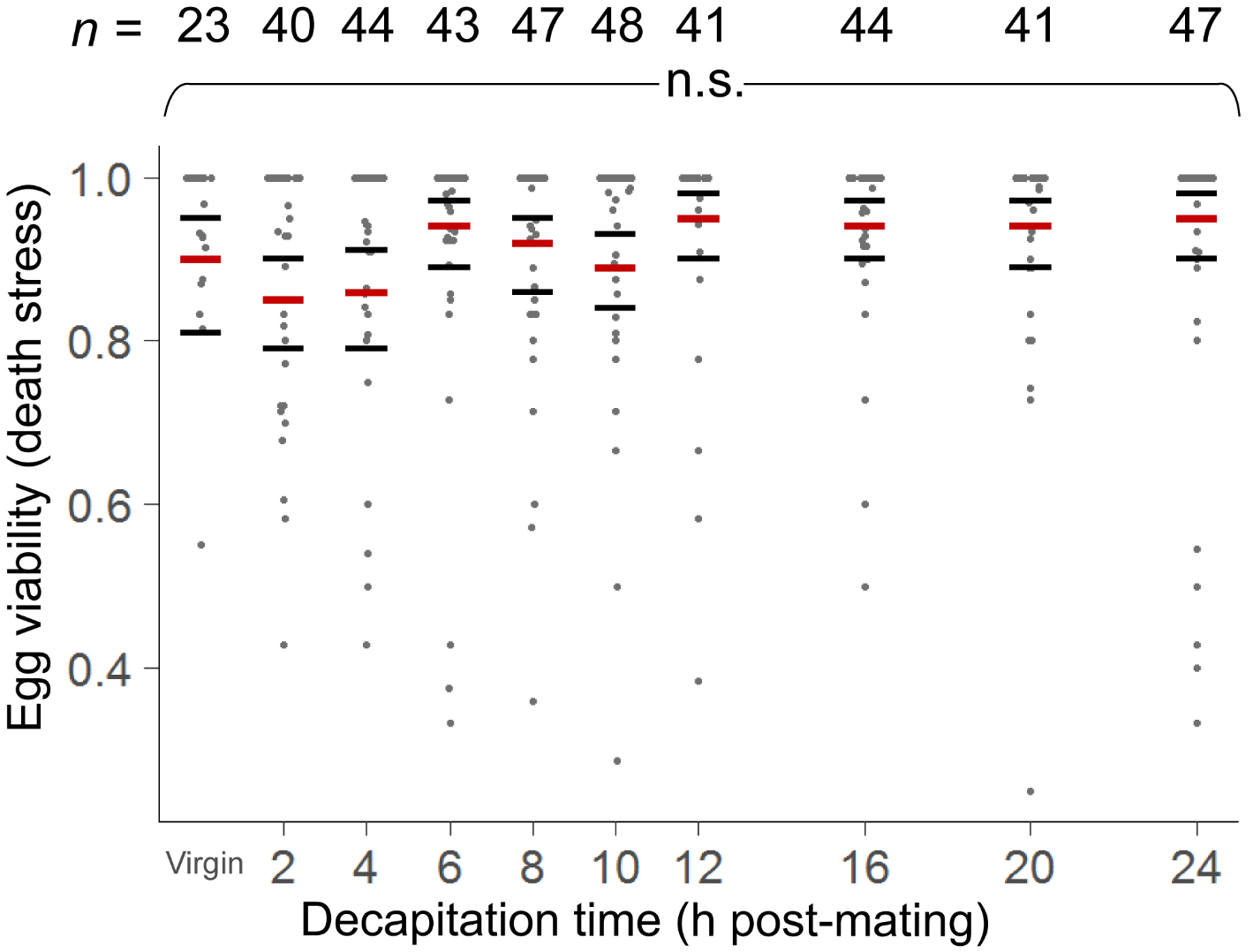


**Supplementary Figure S4.**Viability of eggs laid via death stress oviposition. Each plot includes virgins and females at nine different post-mating intervals. Egg viability was defined as being convex in shape and fully melanized. Viability was significantly predicted by post-mating interval (GLM; *df* = 9,357, *F* = 2.36, *p* = 0.012). However, no difference between intervals remained after Bonferroni multiple comparisons correction. Black bars represent 95% confidence interval, and red bars represent estimated marginal means. Sample sizes same as total eggs laid (Supplementary Fig. S3).

**Supplementary Table S3.** Raw data underlying graphs in Figure 3b, Figure 4, Supplementary Figure S3, Supplementary Figure S4. Spreadsheet includes different tabs for each experiment. Additional information is included in comments on the header of each column.
